# Two Homologous Enzymes of the GalU Family in *Rhodococcus opacus* 1CP—*Ro*GalU1 and *Ro*GalU2

**DOI:** 10.3390/ijms20225809

**Published:** 2019-11-19

**Authors:** Antje Kumpf, Anett Partzsch, André Pollender, Isabel Bento, Dirk Tischler

**Affiliations:** 1 Environmental Microbiology, Institute of Biosciences, TU Bergakademie Freiberg, Leipziger Str. 29, 09599 Freiberg, Germany; anett.partzsch@web.de (A.P.); andre.pollender@ioez.tu-freiberg.de (A.P.); 2 EMBL Hamburg, Notkestr. 85, 22607 Hamburg, Germany; ibento@embl-hamburg.de; 3 Microbial Biotechnology, Faculty of Biology & Biotechnology, Ruhr University Bochum, Universitätsstr. 150, 44780 Bochum, Germany

**Keywords:** glycosylation, UDP-glucose pyrophosphorylase, UDP-glucose, nucleotide donors, *Rhodococcus*, Actinobacteria, gene redundancy, Leloir glycosyltransferases, activated sugar, UTP

## Abstract

Uridine-5’-diphosphate (UDP)-glucose is reported as one of the most versatile building blocks within the metabolism of pro- and eukaryotes. The activated sugar moiety is formed by the enzyme UDP-glucose pyrophosphorylase (GalU). Two homologous enzymes (designated as *Ro*GalU1 and *Ro*GalU2) are encoded by most *Rhodococcus* strains, known for their capability to degrade numerous compounds, but also to synthesize natural products such as trehalose comprising biosurfactants. To evaluate their functionality respective genes of a trehalose biosurfactant producing model organism—*Rhodococcus opacus* 1CP—were cloned and expressed, proteins produced (yield up to 47 mg per L broth) and initially biochemically characterized. In the case of *Ro*GalU2, the *V*_max_ was determined to be 177 U mg^−1^ (uridine-5’-triphosphate (UTP)) and *K*_m_ to be 0.51 mM (UTP), respectively. Like other GalUs this enzyme seems to be rather specific for the substrates UTP and glucose 1-phosphate, as it accepts only dTTP and galactose 1-phoshate in addition, but both with solely 2% residual activity. In comparison to other bacterial GalU enzymes the *Ro*GalU2 was found to be somewhat higher in activity (factor 1.8) even at elevated temperatures. However, *Ro*GalU1 was not obtained in an active form thus it remains enigmatic if this enzyme participates in metabolism.

## 1. Introduction

**Scheme 1:**
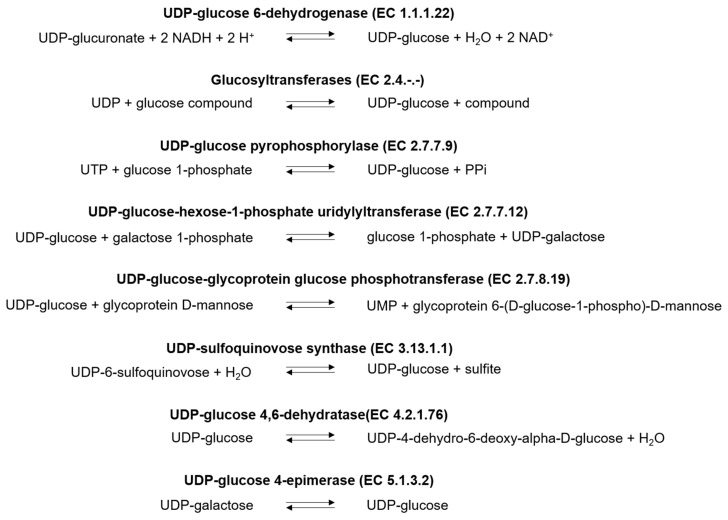
Reactions in which UDP-glucose is consumed or formed. UDP: Uridine-5’-diphosphate, UMP: Uridine-5’-monophosphate, UTP: Uridine-5’-triphosphate, NAD^+^/NADH: Nicotinamide adenine dinucleotide in the oxidized and reduced form.

Uridine-5’-diphosphate (UDP)-glucose is a key metabolite in most organisms and thus used in a variety of reactions of the sugar and starch metabolism, sugar interconversions, amino and nucleotide sugar metabolism, biosynthesis of antibiotics and cell envelope components, and as precursor for different primary and secondary metabolites [1,2,3]. Being an interesting and valuable compound, UDP-glucose attracted more and more attention for biotechnological applications. As an example, in glycosylation reactions, their production and application in fine chemical scale could be shown [1,2,3]. It was presented that whole cell catalysis by sucrose synthase (SuSy) with UDP as precursor led to large-scale production of 100 g_UDP-glucose_/L with a yield of 86% [3]. Another report with 0.1 g/L of free SuSy could achieve 144 g_UDP-glucose_/L with a comparable conversion rate of 85% based on the precursor UDP [1]. The enzymatic production of various sugar-nucleotides can be realized via several UDP-glucose synthesizing enzymes (Scheme 1). Glycosyltransferases, like sucrose synthase, are the most common used representatives [1,4,5,6,7]. Nucleotidyl transferases, like UDP-glucose pyrophosphorylase have been known for a long time but were seldom employed as biocatalysts to produce UDP-glucose, maybe due to low specific activities. Those enzymes are more often studied for their metabolic role.

**Scheme 2:**
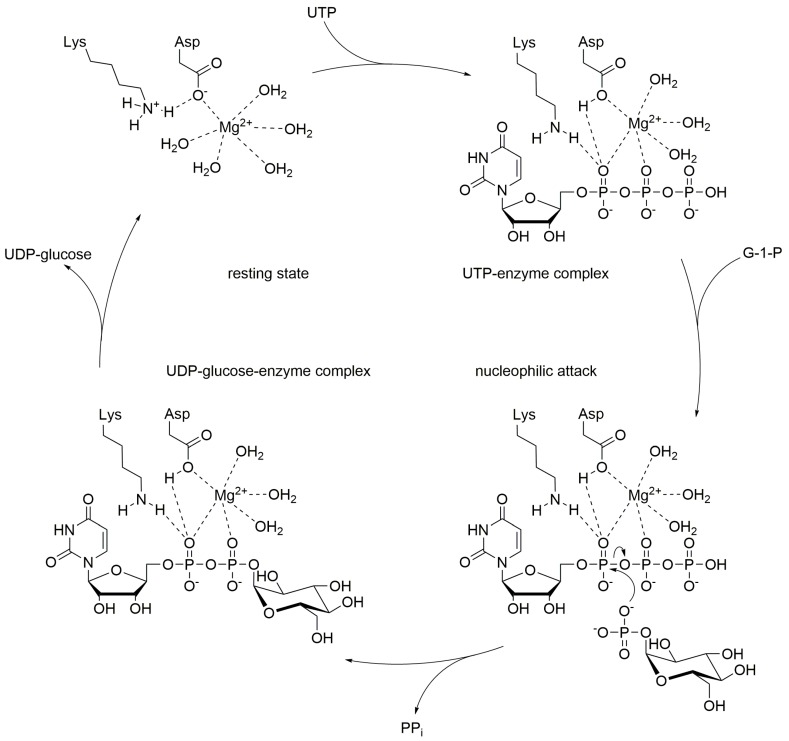
Proposed reaction mechanism of UDP-glucose pyrophosphorylase, adapted from Kim et al. [17].

UDP-glucose pyrophosphorylases (GalU or UGPase; EC 2.7.7.9) catalyze the reversible reaction of glucose 1-phosphate and UTP into UDP-glucose and inorganic pyrophosphate (PP_i_). Enzymes of the GalU family are ubiquitous and can be found among the tree of life [8]. With respect to bacteria mostly proteobacterial GalUs have been studied. Like many other nucleotidyl transferases, also GalU requires divalent cations to promote the reaction (Scheme 2). In most cases magnesium ions are employed, and so far only magnesium chloride has been investigated [9,10,11,12,13,14,15,16]. The reaction mechanism follows a sequential bi-bi-mechanism starting with the binding of UTP to the active site, in presence of a magnesium ion [17], followed by the binding of glucose 1-phosphate. The octahedral coordination sphere of the magnesium positions the substrates in the right way and enables the nucleophilic attack of glucose 1-phosphate on UTP [18]. A lysine, an aspartate and several water molecules within the active site help to stabilize the position of the substrates and cofactor for the proper nucleophilic attack of the phosphoryl oxygen of glucose 1-phosphate towards the α-phosphor atom of UTP [17]. Finally, PP_i_ is released from the GalU/Mg^2+^/UDP-glucose complex [17].

GalU enzymes have been shown to play crucial roles in galactose fermentation [19]. Other metabolic pathways in which this enzyme family is active are similar like the ones UDP-glucose itself plays an important role. It produces UDP-glucose as precursor for sucrose, glucan and amylose in the sugar and starch metabolism. Furthermore, it plays a role in galactose metabolism and glucuronate interconversion, where galactose 1-phosphate is over all converted into UDP-glucuronate with the help of a hexose 1-phosphate uridylyl transferase and a UDP-glucose 6-dehydrogenase. In amino sugar and nucleotide sugar metabolism, GalU is active in interconverting different UDP-sugars via the intermediate UDP-glucose. Additionally, it can be found in glycolipid metabolism and the synthesis of antibiotics. Respectively, many GalU enzymes from several organisms have been investigated to date, e.g., GalUs of plants [20,21,22,23,24], of mammals [25,26,27], including human [28], parasites [29], and many different bacteria [9,10,12,13,30,31,32,33], only to name a few groups. Fields of study included the determination of structures [17,30,34], biochemical characterization [14,31,35,36] and biological function [15,33,37]. 

During those studies, it became clear that many organisms also carry a gene encoding for a GalF, a homologous and putative regulatory protein towards GalU. In *E. coli* the function of the GalF protein was determined by Ebrecht and co-workers [19] to putatively regulate or even interact with GalU while maintaining a low UDP-glucose pyrophosphorylase activity (0.004% of *Ec*GalU). It seems to be an evolutionary artefact, which was observed for several other Enterobacteria, as exemplarily different species and strains of *Klebsiella*, *Yersinia*, *Pectobacterium*, *Shigella*, *Escherichia*, *Pantoea*, *Salmonella*, *Serratia,* and some others [19]. Both proteins, GalU and GalF, seem to have evolved independently from a common ancestor, but only GalU kept the initial enzymatic role. Apparently, GalF underwent several mutations, which led to a much lower activity compared to GalU. By means of mutagenesis of identified key residues, the activity of GalF was restored and comparable to GalU and even a physiological complementation of a GalU-knockout strain was possible [19]. GalUs are known to have two important conserved motifs: The *N*-terminal one, which is involved in the binding of the uracil ring with the sequence G-X-G-T-R-X-L-P-X-T-K (X stands for any amino acid) [8,38] and a V-E-K-P-motif with an important lysine for glucose 1-phosphate binding [38,39,40]. The GalF of *E. coli* was shown to have the same V-E-K-P-motif, but instead of G-L-G-T-R, GalF has G-L-G-M-H residues in the *N*-terminal motif [19]. By means of mutagenesis it could be shown that the alteration of T-R into M-H was possibly the reason for the reduced UDP-glucose pyrophosphorylase activity of *E. coli* GalF. But, the actual function of GalF among Enterobacteria still remains unclear. If gene redundancy and physiological flexibility here between *E. coli* GalU and GalF is of relevance is not yet clarified. Furthermore, it remains enigmatic why this presence of homologous GalU/GalF-proteins has so far only been reported for Enterobacteria among the bacteria. However, in the genomes of *Rhodococcus* species, gene redundancy plays a crucial role [20,22,27,41,42,43]. Interestingly, most *Rhodococcus* species also carry two *galU* genes which we will highlight herein for the first time. Thus, *R. opacus* 1CP encodes for two isoforms of the GalU enzyme—*Ro*GalU1 and *Ro*GalU2, which have not been characterized yet. In some cases, it could be shown that enzymatic isoforms derived from rhodococci have the same or only a slightly different function, so that it was assumed that the coding isogenes have evolved independently from a common ancestor [44]. However, the functionality of respective proteins has not been investigated so far. Furthermore, to the best of our knowledge studies on UDP-glucose pyrophosphorylases in *Rhodocccus* species have not been carried out. Herein we describe the redundant GalU enzymes of *R. opacus* 1CP whereas one was found to be active as recombinant enzyme and might participate in several metabolic pathways, which is discussed in detail. Phylogenetic analysis of the amino acid sequences of GalU proteins of several rhodococci, Enterobacteria, and Actinobacteria helps to further understand the role of *Ro*GalU1 and *Ro*GalU2. This will also give more information on gene redundancy in Actino- and Enterobacteria in general.

## 2. Results

### 2.1. Genome Mining and Phylogenetic Analyses

Via a genome mining approach two genes encoding for two UDP-glucose pyrophosphorylases (GalUs) were identified in *Rhodococcus opacus* 1CP. The respective genes and amino acid sequences used for codon usage optimization were derived from the NCBI protein accession numbers ANS26426 (*Ro*GalU1, theoretical size: 33.2 kDa, GenBank accession number of the codon usage optimized nucleotide sequence: MN617759) and ANS26629 (*Ro*GalU2, theoretical size: 33.9 kDa, GenBank accession number of the codon usage optimized nucleotide sequence: MN617760). They have a sequence identity of more than 70% on amino acid level to each other when compared over full length. 

Having a look at the genomic environment of both genes, various genes were found flanking *RogalU1*, which were involved in the sugar- and nucleotide metabolism, e.g., a mannose 6-phosphate isomerase, a D-glycero-D-manno-heptose 1-phosphate guanosyltransferase or a undecaprenyl-phosphate galactose phosphotransferase, whereas *RogalU2* is flanked by cation channels, heat shock proteins or putative regulatory proteins.

**Figure 1:**
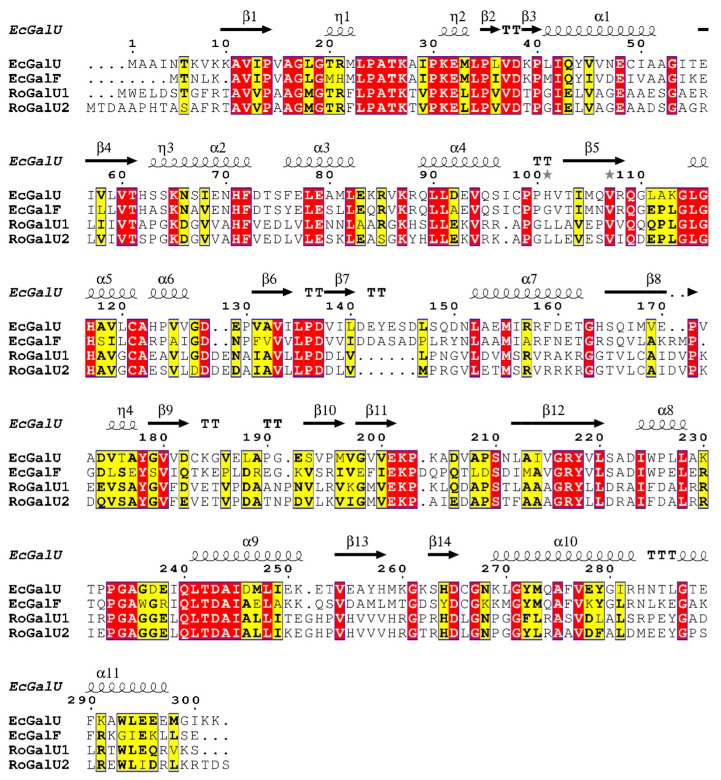
Alignment of *Ro*GalU1 and *Ro*GalU2 with the GalU and GalF amino acid sequences of *E. coli* K-12 [19], executed with the MUSCLE algorithm [45,46] in the program MEGA X [47] for multiple sequence alignment with default settings and imaged with ESPript 3.0 [48]. Conserved amino acids are color-coded. Red box with white letters: strictly identical amino acids, yellow box with black bold letters: similar amino acids, which are conserved among at least two sequences, black squiggles with α or η signs: α-helical structures, black arrows with β sign: β-sheet structures, **TT**: strict β-turns, **TTT**: strict α-turns, grey stars: residues with alternate conformations.

Analyzing the amino acid sequences of the GalUs originating from *R. opacus* 1CP, both contain the same motifs for uracil binding, G-X-G-T-R-F-L-P (start: amino acid 17 in *Ec*GalU sequence; 18 and 21 in *Ro*GalU1 and *Ro*GalU2, respectively). Equally, both display the V-E-K-P motif (start: amino acid 200 in *Ec*GalU sequence; 199 and 202 in *Ro*GalU1 and *Ro*GalU2, respectively) with the important lysine (K202 in *Ec*GalU) for binding of glucose 1-phosphate (see Figure 1; the subsequent numbering of amino acids is according to *Ec*GalU; [19]). Interestingly, only the GalF of *E. coli* shows an alteration from T20-R21 to M-H in the first motif, which was shown before to be the reason for the reduced UDP-glucose pyrophosphorylase activity of *E. coli* GalF. Large parts of *Ro*GalU1 and *Ro*GalU2 are identical and seem to be conserved. Several of those conserved amino acids can be found in *Ec*GalU and/or *Ec*GalF, respectively. Furthermore, all four sequences share the same residues for the hydrogen bonding of the uracil ring (A16-G17 and Q109), and at least the *Rhodococcus* GalUs and *Ec*GalU have the same amino acids for the phosphoryl binding of UDP-glucose (G179, K202 and G-A-G-D, start: amino acid: 234). The overall alignment indicates that the structure of all four proteins should be highly similar, as the secondary elements presented by α-helical and β-sheet areas of *Ec*GalU are present in *Ro*GalU1 and *Ro*GalU2, as well.

**Figure 2:**
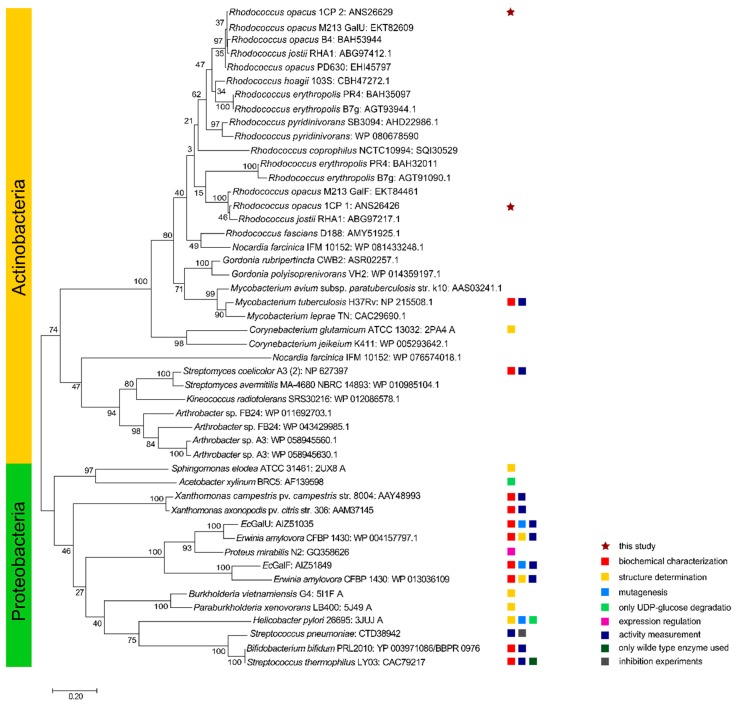
Phylogenetic tree of *Ro*GalU1 and *Ro*GalU2 with similar characterized or related UDP-glucose pyrophosphorylase sequences. The evolutionary history was inferred by using the Maximum Likelihood method and JTT matrix-based model [49]. The tree with the highest log likelihood (−13775.30) is shown. The percentage of trees in which the associated taxa clustered together is shown next to the branches. Initial tree(s) for the heuristic search were obtained automatically by applying Neighbor-Join and BioNJ algorithms to a matrix of pairwise distances estimated using a JTT model, and then selecting the topology with superior log likelihood value. The tree is drawn to scale, with branch lengths measuring the number of substitutions per site. This analysis involved 48 amino acid sequences. There was a total of 375 positions in the final dataset. Evolutionary analyses were conducted in MEGA X [47].

The alignment (Figure 1; part of the complete alignment with all 48 amino acid sequences) that underlies the phylogenetic tree (Figure 2) shows remarkable sequence similarities and again huge conserved areas between the GalU sequences of different actinobacterial species and especially strains of *Rhodococcus*, *Mycobacterium*, *Corynebacterium,* and *Gordonia*. As they are close relatives, it is not surprising that those sequences cluster together. It is striking that there are two sequence sections around the G-X-G-T-R-F-L-P (start: amino acid 17) motif and the V-E-K-P (start: amino acid 200) motif, which are highly conserved among all chosen sequences. Besides those and the highly conserved motifs for binding of the substrates and products, there are also three more conserved motifs: One G-L-G-H sequence (start: amino acid 114) before and two sequences between the V-E-K-P motif and the *C*-terminus, namely G-R-Y-L-L (start: amino acid 216) and Q-L-T-D-A-I (start: amino acid 240) within all analyzed sequences. In addition, those seem to be highly conserved, not only among Actinobacteria, but also Enterobacteria. Furthermore, the sequences contain single conserved amino acids, like G53, F72, E79, D137, Y178, S210, G261, D265, and G267 (Figure 1). 

The above described alignment was used and completed by numerous GalU protein sequences in order to generate a distance tree for a phylogenetic analysis (Figure 2). The first big branch of the tree contains all actinobacterial proteins, which are very close relatives. Within those, also *Rhodococcus* enzymes form one big cluster. Interestingly, the enzymes from the same strains do not cluster together, but form two different smaller clusters. The *Rhodococcus* strains containing only one GalU protein are mapped within the branch of our tested and active *Ro*GalU2. The second big branch of the tree contains Actinobacteria in the upper part, Firmicutes, and Proteobacteria in the lower part.

### 2.2. Recombinant Expression of RogalU1 in E. coli, Purification, and Renaturation

**Figure 3:**
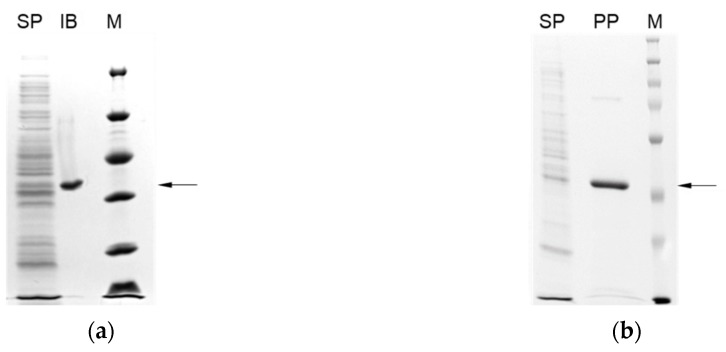
SDS-PAGE gels of *Ro*GalU1 (**a**) and *Ro*GalU2 (**b**). Arrows indicate the expected molecular weight of 33 kDa for *Ro*GalU1 and 34 kDa for *Ro*GalU2. SP: soluble proteins, IB: inclusion bodies, PP: purified protein, M: Marker.

For characterization purposes in more detail *RogalU1* was cloned into the expression vector pET16bP. After transformation of chemically competent *E. coli* BL21(DE3) pLysS with pET16bP-*RogalU1* overexpression and respective protein production and purification were carried out as described in the material and methods section. The SDS-PAGE in Figure 3a shows the expected molecular weight of *Ro*GalU1 of 33 kDa, but most of the produced enzyme is present as insoluble protein in the cells. The formation of those inclusion bodies was determined from the greyish to white color of the pellet after crude cell extract preparation. Hence, it was tried to renature the misfolded protein with the Thermo Scientific Pierce Protein Refolding Kit according to the manufacturer’s instruction. Unfortunately, it was not possible to obtain *Ro*GalU1 in an active and soluble form. Also, protein within the inclusion bodies only had a marginal residual enzyme activity. This was determined to be <0.001 U mg^−1^ by means of the standard test which is described in the materials and methods section (4.6). In this work, we determined throughout enzyme activity with respect to product formation (1 U corresponds to the formation of 1 µmol UDP-glucose per minute). In order to turn inclusion bodies into native protein, the expression was altered with respect to various media and cultivation conditions. All efforts failed and thus this enzyme was not characterized further.

### 2.3. Recombinant Expression in E. coli, Purification and Production of Active RoGalU2

The gene *RogalU2* was treated as described in the materials and methods and similarly as the *RogalU1* described above. In this case, it was possible to produce about 47 mg of pure, soluble protein with an expected molecular weight of 34 kDa (see Figure 3b) from 25 g wet biomass. The maximum enzyme activity observed was 3.6 U mg^−1^ in the direction of UDP-glucose formation, using the standard reaction conditions. In addition, different media for gene expression were tested (LB, TB, or NB), but no significant difference in terms of protein yield or activity was found. TB medium was the fastest medium for cell growth and led to most biomass, therefore using TB auto induction medium for later large-scale production was an obvious choice.

**Figure 4:**
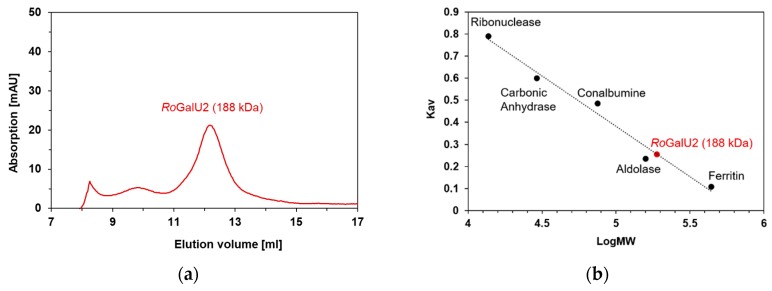
Size exclusion chromatography of *Ro*GalU2 (**a**) and the corresponding calibration measurements (**b**). The calibration curve was prepared with the following proteins of known molecular weight (given in brackets): Ribonuclease (13.7 kDa), Carbonic Anhydrase (29 kDa), Conalbumin (75 kDa), Aldolase (158 kDa) and Ferritin (440 kDa). All elutions were performed in a buffer containing 50 mM Hepes, pH 7.0 and 1 mM magnesium chloride. See methods Section 4.5. The protein concentration was either 0.2 or 2.0 mg mL^−1^ whereas the latter is presented herein.

In order to validate the oligomeric state of the enzyme, a size exclusion chromatography under non-denaturizing conditions was performed in duplicates with two different concentrations of the protein (0.2 and 2.0 mg mL^−1^), each. The elution profiles were similar in those cases (not shown). The respective calibration was also measured twice and revealed the occurrence of this protein mostly in a hexameric state (188 kDa elution volume vs. subunit size of 34 kDa; Figure 3 and Figure 4). Thus, we can state that under these conditions the protein was present mostly in a single hydrodynamic state and therefore protein characterization can be performed.

### 2.4. Determining Optimum Reaction Conditions for RoGalU2

**Figure 5:**
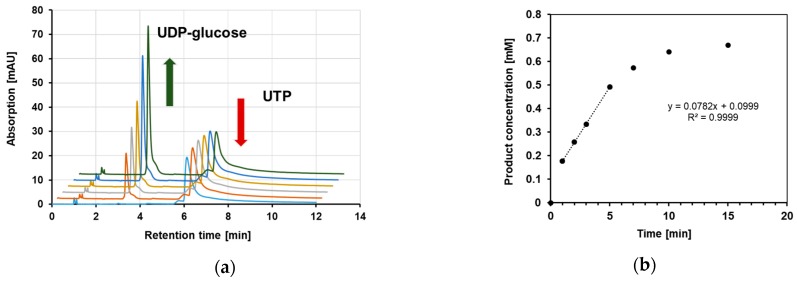
Enzymatic conversion of UTP and glucose 1-phosphate into UDP-glucose and PP_i_. In (**a**) the HPLC chromatograms of a time course from 0 to 7 min are presented and the determined concentration of UDP-glucose was used to calculate a product formation rate as shown in (**b**). For this analysis the standard enzyme assay and determination methods as described in materials and methods section have been used.

In order to collect proper kinetic data for the enzyme activity of *Ro*GalU2 in the direction of UDP-glucose formation, it was necessary to find optimum reaction conditions. In addition, the product detection method and fitting procedure had to be established. UDP-glucose formation by *Ro*GalU2 was determined by HPLC as described in materials and methods section. A typical result and a respective rate determination is presented in Figure 5.

**Figure 6:**
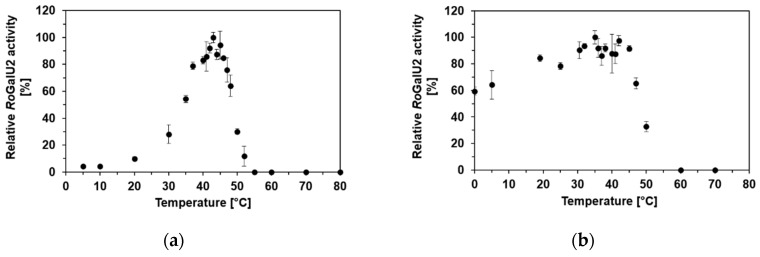
Temperature-dependent activity (**a**) and stability (**b**) of purified *Ro*GalU2 enzyme was measured in triplicates. Activity was measured with pre-incubation of the assay mixture (2 mM UTP, 2 mM glucose 1-phosphate, 4 mM MgCl_2_, 50 mM Hepes buffer, pH 7.0, 30 µg *Ro*GalU2, 1 mL reaction volume) at indicated temperatures. The enzyme was stored on ice and directly used for measurements. For stability measurements the enzyme was incubated shaking for 30 min at indicated temperatures and directly used for activity assay at 45 °C. Means and standard deviations are shown. 100% relative *Ro*GalU2 activity correspond to 6.7 U mg^−1^ ((**a**), activity) and 5.0 U mg^−1^ ((**b**), stability), respectively.

Firstly, the reaction temperature was varied from 4 °C to 87 °C. There was an activity plateau determined between 40 °C and 45 °C, with a maximum observed enzyme activity of 6.7 U mg^−1^ (Figure 6a). To monitor the stability of *Ro*GalU2 at different temperatures, the enzyme was incubated for 30 min at temperatures ranging from 0 °C to 70 °C. The activity assay was then carried out at 45 °C. *Ro*GalU2 shows at least 80% of activity when incubated for 30 min between 19 °C and 45 °C (see Figure 6b). Because of the higher activity that could be achieved at 42 °C *versus* 45 °C, it was decided to perform all subsequent experiments at 42 °C.

**Figure 7:**
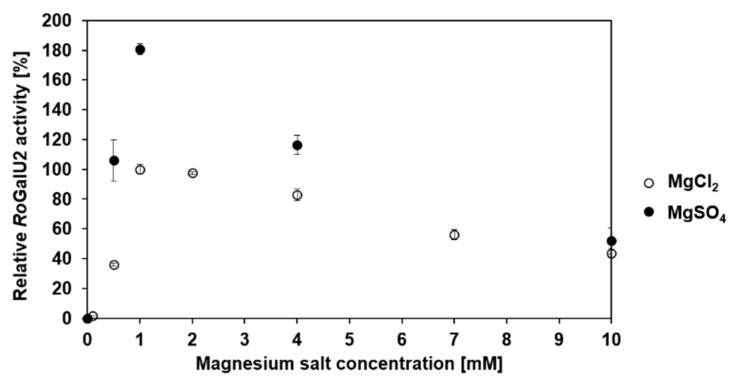
Relative *Ro*GalU2 enzyme activity depending on the MgCl_2_ and MgSO_4_ concentration between 0 mM and 10 mM. Assay mixture contained 2 mM UTP, 2 mM glucose 1-phosphate, 0–10 mM MgCl_2_ or MgSO_4_, 50 mM Hepes buffer, pH 7.0, 30 µg *Ro*GalU2. Reaction was carried out at 42 °C in 1 mL reaction volume. Means with standard deviations of triplicate measurements are shown. 100% relative *Ro*GalU2 activity correspond to 5.2 U mg^−1^.

To verify the dependence of *Ro*GalU2 on magnesium the concentration of magnesium chloride and magnesium sulfate present in the standard reaction setup at 42 °C was varied between 0–10 mM. For magnesium salt concentrations below 1 mM, the standard buffer of the enzyme had to be exchanged by a buffer without magnesium chloride freshly prior to respective experiments since *Ro*GalU2 is not stable in a buffer without magnesium. The activity was also measured after every buffer exchange. Figure 7 shows the absolute dependence of the activity on the concentration of magnesium ions. There is no activity and thus UDP-glucose formation measurable without magnesium salt. The highest activity was determined to be 5.2 U mg^−1^ with magnesium chloride and 9.5 U mg^−1^ with magnesium sulfate, in the presence of only 1 mM of the respective salt. When increasing the magnesium concentration, the *Ro*GalU2 activity decreases significantly, falling below 60% with 10 mM magnesium salt. Thus, it can be concluded that magnesium is a crucial cofactor for the enzyme as presented in Scheme 2, respectively.

The activity of *Ro*GalU2 with respect to magnesium salts was most similar at a concentration of 4 mM. Thus, further divalent cations were tested as metal chloride salts at 4 mM each like manganese, nickel, cobalt, calcium, and zinc. In addition, trivalent metal cations like iron and aluminum were tested. No activity was observed with the trivalent metal chlorides. For all other divalent chlorides, the relative activity was below 20%. Thus, a magnesium dependency was clearly determined. 

To test whether masking of the magnesium by means of EDTA results in a reduced enzyme activity, we applied 1 mM EDTA to the reaction solution and could not observe any activity. Therefore, the standard assay was used but at a lower magnesium chloride concentration, which was set to 1 mM as well. We also tested the reaction in presence of acetonitrile as solvent and received the same result. This was of importance since acetonitrile was used to stop the reactions prior to HPLC analysis as well as to adjust samples towards elution conditions. Thus, it was proven that acetonitrile was a proper reagent to stop the reaction.

**Figure 8:**
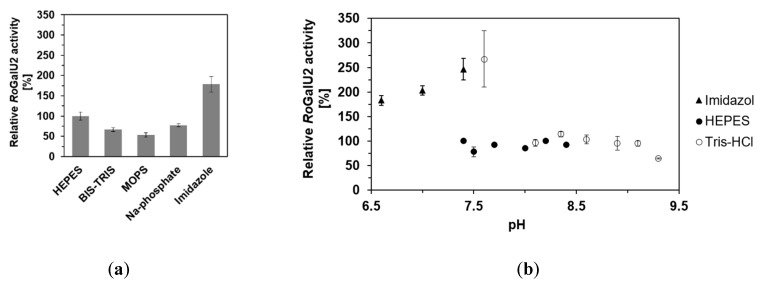
*Ro*GalU2 enzyme activity depending on the buffer (**a**) and pH (**b**) of the reaction solution. (**a**) Assay mixtures contained 1.5 mM UTP, 250 mM glucose 1-phosphate, 1 mM MgCl_2_, 50 mM buffer, pH 7.4, 1.7 µg *Ro*GalU2. Reaction was carried out at 42 °C in 1 mL volume. (**b**) Assay mixtures contained 1.5 mM UTP, 250 mM glucose 1-phosphate, 1 mM MgCl_2_, 50 mM Imidazole, pH 6.6–7.4 (black triangle) or 50 mM Hepes, pH 7.4–8.4 (black circle) or 50 mM Tris-HCl, pH 7.6–9.3 (white circle), 1.7 µg *Ro*GalU2. Reaction was carried out at 42 °C in 1 mL volume. Means with standard deviations are shown out of a minimum set of three independent measurements. 100% relative activities correspond to 126 U mg^−1^ (**a**) and 109 U mg^−1^ (**b**).

Different buffers like Bis-Tris, MOPS, sodium phosphate and imidazole were also tested to see whether the enzymatic activity can be further increased. The pH of the reaction solution was also varied ranging from 6.6–9.3 in total and split into the following ranges of different buffers: 6.6–7.4 (Imidazole), 7.4–8.4 (Hepes), and 7.6–9.3 (Tris-HCl). Figure 8 shows a graph with the comparison of *Ro*GalU2 activities measured in those reaction solutions.

In none of the used buffers the relative *Ro*GalU2 activity dropped below 60%. But it was possible to increase the relative activity up to 180% with Imidazole at pH 7.4. The pH profile even shows an increase up to 250% in activity when Imidazole or Tris-HCl are used at pH values of around 7.5. Interestingly, there is no significant difference when Hepes buffer is used at pH values between 7.4 and 8.3 or when Tris-HCl is used between pH 8.1 and 9.1. Here we only observed relative *Ro*GalU2 activities around 80%. 

The above described results allowed to formulate an improved enzyme assay with the following conditions: 1.5 mM UTP, 250 mM glucose 1-phosphate, 1 mM magnesium chloride, 50 mM Hepes, pH 7.4, 42 °C in 1 mL reaction volume. Even though the activity of *Ro*GalU2 is higher with Imidazole pH 6.6–7.4 or Tris-HCl pH 7.6, we decided to continue using Hepes pH 7.4, because the standard deviation with Hepes buffer was found to be much smaller than with other buffers. This might be due to the weaker buffering properties of Tris-HCl or Imidazole in the applied range.

### 2.5. Enzyme Kinetics with RoGalU2

**Figure 9:**
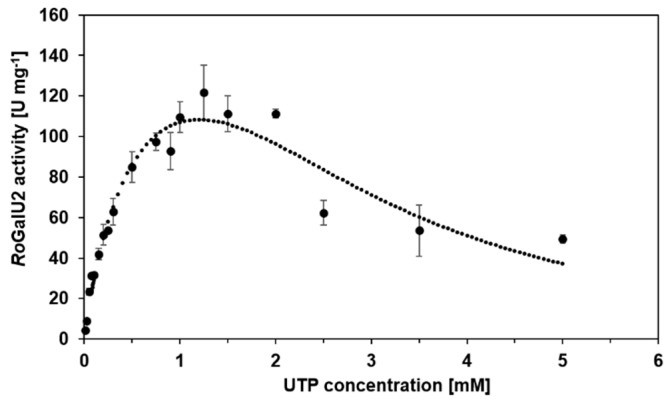
*Ro*GalU2 enzyme activity depending on the UTP concentration between 10 µM and 5 mM at a fixed glucose 1-phosphate concentration of 250 mM. Fit is calculated by the Model of Yano and Koga [50], for enzyme kinetics with substrate inhibition. The assay mixture contained 0.01–5 mM UTP, 250 mM glucose 1-phosphate, 1 mM MgCl_2_, 50 mM Hepes buffer, pH 7.4, 1.7 µg *Ro*GalU2. Reaction was carried out at 42 °C in 1 mL volume. Mean values with standard deviations from triplicates are shown.

In order to collect proper kinetic data, the above described improved enzyme assay was employed. Thus, we could now determine the common kinetic constants *K*_m_, *V*_max_, *k*_cat_, and *k*_cat_/*K*_m_ by means of varying the substrate concentrations. Already here we like to state the *K*_m_ and thus *k*_cat_ values have to be considered as apparent values as later indicated in Table 1. This is necessary as a saturation of the enzyme by both substrates cannot be secured as the later on presented data show. Pre-experiments defined 1.5 mM UTP and 250 mM of glucose 1-phosphate as most suitable fixed concentrations for the variation of the opposite substrate, respectively. As shown in Figure 9 UTP was varied between 10 µM and 5 mM with a fixed concentration of 250 mM glucose 1-phosphate. The data were fitted to the model of Yano and Koga [50] (Equation (2), see Methods section) for substrate inhibition, because above 1.5 mM UTP a strong substrate inhibition became obvious. Below this concentration, the *Ro*GalU2-activity reaches a maximum observed activity of about 122 U mg^−1^. The calculated *V*_max_ was determined to be 177 U mg^−1^ according to the inhibition fit of Yano and Koga [50].

**Figure 10:**
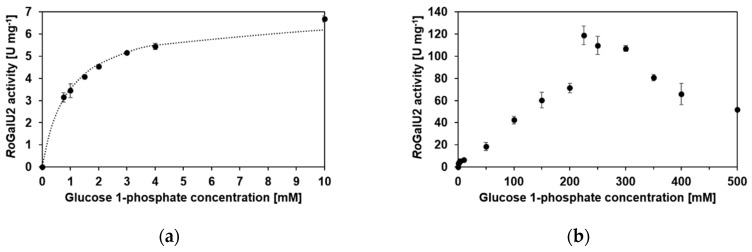
*Ro*GalU2 enzyme activity depending on the glucose 1-phosphate concentration between 50 µM and 500 mM at a fixed UTP concentration of 1.5 mM. Data for glucose 1-phosphate concentrations between 0 and 10 mM were fit to the kinetic model of Michaelis–Menten (**a**). The data obtained over the total glucose 1-phosphate variation are presented in (**b**), whereas no fit was able to describe the data. Assay mixture contained 0.05–500 mM glucose 1-phosphate, 1.5 mM UTP, 1 mM MgCl_2_, 50 mM Hepes buffer, pH 7.4, 1.7 µg *Ro*GalU2. Reaction was carried out at 42 °C in 1 mL volume. Means with standard deviations are shown from a minimum of triplicates.

Figure 10 shows the graphs for substrate variation of glucose 1-phosphate between 50 µM and 500 mM with 1.5 mM UTP in each reaction. Figure 10a shows a Michaelis–Menten-like kinetic at concentrations between 50 µM and 10 mM. This is common for many GalU enzymes as discussed later. But with a closer look at the data, it became clear that the model does not describe the values in a proper way. In contrast to common Michaelis–Menten kinetics, the *Ro*GalU2 activity still increased, beyond the concentration of 10 mM glucose 1-phosphate applied. Therefore, the collection of data for higher glucose 1-phosphate concentrations was necessary and showed a kinetic behavior that was different from general models that describe enzyme kinetics (Figure 10b), with a maximum observed activity of about 119 U mg^−1^. No model allowed to fit the obtained data properly. Further, we repeated the experiments with other batches of protein and the results were similar. To achieve a maximum formation rate of UDP-glucose, a high concentration of glucose 1-phosphate was mandatory, resulting in those data.

The kinetic properties of *Ro*GalU2 are summarized in Table 1. Further, to describe the substrate scope of *Ro*GalU2, we used UTP, ATP, GTP, CTP, and dTTP as nucleotides and glucose 1-phosphate, galactose 1-phosphate, ribose 5-phosphate and glucose as sugars in different combinations to each other. Only with sugar 1-phosphates and UTP or dTTP, product formation was detectable. Nevertheless, the relative activity of the reaction of dTTP and glucose 1-phosphate could not be evaluated due to unavailability of a product standard. A low relative *Ro*GalU2 activity of 2% was observable when galactose 1-phosphate and UTP were tested (100% relative *Ro*GalU2 activity corresponded to 8.1 U mg^−1^). With other nucleotides or sugars no activity or product formation was observable.

## 3. Discussion

UDP-glucose pyrophosphorylases are important enzymes for the general metabolism of many organisms as highlighted in the introduction. However, among the bacterial representatives mostly GalU enzymes from Proteobacteria have been studied. To a lesser extent, GalUs of Actinobacteria were described, as for example some information are available for the ones originating of *Mycobacterium* or *Streptomyces* species [10,13,51]. Here we wanted to add knowledge and studied the enzyme family from rhodococci, which is interesting from a phylogenetic as well as mechanistic point of view. In a model organism, *Rhodococcus opacus* 1CP, relevant for trehalose biosurfactant production or aromatic compound degradation [52,53], two isogenes encoding for UDP-glucose pyrophosphorylases were identified and described for the first time herein.

A phylogenetic tree based on an alignment of 48 protein sequences of partially characterized UDP-glucose pyrophosphorylases was generated (Figure 2). The tree is separated into two groups; proteobacterial and actinobacterial representatives. It should be stressed that some protein sequences have been annotated as GalU and others as GalF enzymes or regulatory proteins, respectively, but in most cases this has not been experimentally verified.

The first group contains GalU- or GalF-designated protein sequences of partially characterized UDP-glucose pyrophosphorylases or related sequences from Actinobacteria, the other contains GalUs and GalFs from Proteobacteria. The branch of actinobacterial enzymes is separated again into two subgroups; Corynebacteriales and other orders of Actinobacteria. Within the branch of corynebacterial enzymes different *Rhodococcus* species cluster together. Gene products derived from soil or wastewater populating rhodococci (which encode for two GalU enzymes) cluster into two subbranches, respectively. Within those, GalUs of the zoonotic pathogen or animal infecting *Rhodococcus* species, such as *R. coprophilus* and *R. hoagie* (*equii*), as well as those *Rhodococcus* strains that only comprise a single GalU gene form one cluster. Herein, the protein *Ro*GalU2 from our model strain is localized. An exception is the plant pathogenic *R. fascians*, which also encodes for only one GalU protein. It seems more related to a pathogenic *Nocardia* then to other rhodococci enzymes. From those *Rhodococcus* species carrying two GalUs, a second branch is formed in which the protein *Ro*GalU1 clusters. Another branch within corynebacterial representatives is formed by GalUs of the genera *Gordonia*, *Mycobacterium* and *Corynebacterium*. And another more distant group of the actinobacterial GalUs contains enzymes of the genera *Streptomyces*, *Kineococcus* and *Arthrobacter*. Interestingly, *Arthrobacter* also has strains carrying two isoforms of GalU proteins, but they form a group far from the *Rhodococcus* proteins. A similar behavior is present in the group of proteobacterial GalUs including some annotated but also experimentally verified GalFs. Here the enzymes are arranged according to their phylogenetic relation as expected. However, *E. coli* and *Erwinia amylovora* each encode two related proteins which form distinct branches. 

As the branch of proteins named GalU2 also contains other actinobacterial GalUs only having one GalU isoform, it is possible that GalU2 is the older protein with the initial function in the genus *Rhodococcus*. Thus, GalU1 might have evolved from GalU2 and gained the function of a UDP-glucose pyrophosphorylase. But the genetic environment of the respective coding genes does not support this hypothesis. The genetic environment of *RogalU1* is more related to the sugar metabolism with sugar-phospho transferases and sugar-phospho isomerases, whereas the genetic environment of *RogalU2* comprises many putative and for the sugar or amino metabolism unspecific genes. It consists of different genes coding for cation and phosphate channels, transmembrane proteins, conductance and mechanosensitive channels, acting as an osmotic release valve in response to osmotic stress, as well as ATP converting enzymes and heat shock proteins. Interestingly, the gene cluster of *RogalU1* is only available in *R. jostii* RHA1, whereas the gene cluster surrounding *RogalU2* is present in many other Actinobacteria, like *N. farcinica*, *M. smegmatis*, *M. tuberculosis*, *C. glutamicum*, among others. Therefore, the *RogalU2* cluster seems to be the more common one for Actinobacteria.

Interestingly, *Ro*GalU2 has 99% amino acid sequence identity to the GalU regulator GalF of *R. opacus* PD630. However, this GalF designation for strain PD630 is based only on a bioinformatic annotation and no other *galU* gene is present in the genome of *R. opacus* PD630. Thus, this protein must also be an UDP-glucose pyrophosphorylase and not a regulatory element. *Ro*GalU1 only has 83% sequence identity to this mentioned protein of *R. opacus* PD630. Thus, it seems likely that the protein more related to our *Ro*GalU2 is of importance for rhodococci, which will need to be verified by further studies and representatives. GalF of *E. coli* only has a slight residual UDP-glucose pyrophosphorylase activity. *E. coli* GalF showed a drastically increased activity and substrate specificity for glucose 1-phosphate when exchanging the methionine in position 15 into a threonine and the histidine in position 16 into an arginine (both within the G-X-G-T-R-F-P-L motif of GalUs), which are the corresponding residues in *E. coli* GalU [19]. In the sequences of *Ro*GalU1 and *Ro*GalU2, there is no alteration in the G-X-G-T-R-F-L-P motif. Thus, a regulatory role among the *Rhodococcus* proteins is excluded at this stage.

The underlying alignment of the distance tree shows that GalU enzymes in general are very conserved among bacteria, and that the degree of conservation within Actinobacteria is even higher. The first 100 amino acids are almost fully conserved with only some exceptions. As the two largest conserved areas of all shown sequences are around the motifs for substrate and product binding, it is obvious that the reaction mechanism is very likely the same in all bacterial GalU enzymes. It is striking that there are also four more motifs and several single amino acids which are highly conserved among bacterial GalU sequences. The tyrosine of the G-R-Y-L-L motif and the leucine in the Q-L-T-D-A motif are also mentioned by Aragão et al. to form a hydrophobic cap to the base of the sugar ring in the active site [9]. It could be possible that the other motifs and amino acids are not only important for the structure formation, like establishing the subunit interaction, but also for the construction of the active site, the binding of substrate and product or even the binding of magnesium ions as cofactors. They could help to stabilize the nucleophilic attack of glucose 1-phosphate to UTP or to bring the active site in the right conformation when the substrates are bound.

Hence, both genes *RogalU1* and *RogalU2* were successfully cloned into expression systems and protein production for a subsequent biochemical characterization was studied. It was not possible to produce active and soluble *Ro*GalU1. In addition, the inclusion bodies obtained had only a low residual enzyme activity. Neither the optimization of medium or expression conditions nor the renaturation of wrongly folded protein lead to soluble and active *Ro*GalU1. Inclusion bodies can be avoided or reduced by different methods. Reduction of the expression temperature to 14–16 °C or even lower, reduction of the expression time or ITPG concentration could possibly increase the amount of soluble active protein [54]. Also, co-expression of the gene of interest with chaperones, are reported to help folding the protein in the right way [55]. The production of a fusion protein with mCherry or other suitable proteins can increase the protein solubility as well [2]. The sole production of inclusion bodies of a GalU from *M. tuberculosis* could be overcome by using an expression system which was more related to the donor organism, so gene expression was achieved in competent *M. smegmatis* cells and the mycobacterial specific expression vector pMIP12 for improved gene expression [51]. 

*Ro*GalU2 was successfully overproduced and purified in reasonable amounts compared to literature, but with a higher yield of insoluble recombinant *Ro*GalU2, as well [13]. SDS-PAGE revealed the expected molecular weight of about 34 kDa. Within bacteria this molecular weight corresponds to that of other GalUs [12,13,56]. The oligomeric state of the protein was determined to be hexameric by size-exclusion chromatography. But the other known bacterial GalUs were shown to be dimers [10,31] or tetramers [8,9,12,17,30]. Only Lai et al. reported the GalU of *M. tuberculosis* strain H37Rv to be dimeric and hexameric in solution [13]. As the latter one is closely related to our target *Ro*GalU2 this fits well to our findings (Figure 2). But we did observe a hexameric state under applied conditions, which can be studied in more detail by altering the buffer or presence of divalent ions through the size exclusion chromatography. Respectively, the presence of a protein in a single hydrodynamic state allowed us to study the biochemical properties in more detail.

*Ro*GalU2 with a *V*_max_ of 177 U mg^−1^ (UTP) and 119 U mg^−1^ (G1P), respectively, has a high activity compared to other bacterial GalUs. Many other bacterial GalUs show activities between 0.1 U mg^−1^ for *M. tuberculosis* GalU [13] and 90 U mg^−1^ for *Xantomonas campestris* [31]. Similar or slightly higher activities for bacterial GalUs are known as well, like 270 U mg^−1^ for *Streptomyces coelicolor* GalU [10] and 340 U mg^−1^ for *E. coli* GalU [19]. Higher activities are only known from eukaryotes [24,57,58]. *Ro*GalU2 also showed a high apparent turnover frequency (app. *k*_cat_) with 96 s^−1^. But this value is very heterogeneous among bacteria, showing lower, similar and higher turnover frequencies [13,19,30]. An overview on bacterial GalU enzymes is provided in Table 2.

The maximum activity was observed between 40 °C and 45 °C and the protein was stable up to 45 °C. The enzymatic activity drastically decreased after an incubation for 30 min at higher temperatures. Other GalUs have temperature optima around only 37 °C [60,61]. Only nucleotidyltransfrases of thermophilic bacteria and Archaea have a higher temperature optimum [62,63] and a half-life of 30 min at 95 °C [36]. However, *Ro*GalU2 originates from a mesophilic Actinobacterium, *R. opacus* 1CP, which grows best at 30 °C. Thus, this temperature optimum was not expected for a metabolically relevant enzyme. Only two other enzymes of this strain showed a higher temperature activity or stability so far; a flavin-dependent monooxygenase [64] and membrane-linked isomerase [65]. 

The use of magnesium sulfate with respect to UDP-glucose pyrophosphorylases was not described in literature before. Here we show the definite dependency on magnesium ions and an activity increase to 180% when using sulfate as anion instead of chloride. The total electronegativity of sulfate is higher than of chloride, which could be advantageous for charge neutralization and coordination of the phosphoryl oxygen. This could then help binding UTP and implementing the nucleophilic attack, which is the base for the proposed reaction mechanism (Scheme 2) [17,18]. Another possibility is that the sulfate variant of magnesium salt forms a more stable complex with UTP than the chloride. As Kleczkowski already reported, those complexes could be the actual substrates for GalUs and that free UTP inhibits the reaction when present in too high concentrations [66]. This is in agreement to our kinetic study in which substrate inhibition was determined at higher UTP concentrations (Figure 9). Furthermore, this is supported by the results obtained by the variation of magnesium salt concentration. There the activity of *Ro*GalU2 decreased rapidly when the concentration of the magnesium salt was higher than 1–2 mM. It is likely that the activity increases when both, the cofactor and UTP, are fed in equimolar concentrations. But an experimental set up will need to verify this hypothesis. Other groups did not report the occurrence of substrate inhibition. Here, we describe a substrate inhibition for both, glucose 1-phosphate and UTP. The only report showing a similar behavior of a GalU enzyme at increasing glucose 1-phosphate concentrations is for the enzyme from potato by Gupta and colleagues [57]. But it has to be noted that the tested glucose 1-phosphate concentration range was quite small (0.05–1 mM) and the activity still increased at 1 mM [57]. 

GalUs show activities with the same divalent metal ions that were tested with *Ro*GalU2 and also the same degree of inhibition by EDTA [12,36,63,67]. Thus, a clear preference for Mg^2+^ is demonstrated for all GalU enzymes.

The literature reported pH range tolerated by GalUs is huge, between pH 5.5 and pH 10.0, but the optimum is often found around pH 7.5 [13,36,63,67]. *Ro*GalU2 behaves expectedly with a pH optimum of around 7.5, depending on the buffer. However, it maintains a comparable high activity up to a pH of about 9 and thus behaves as many GalU enzymes. 

*Ro*GalU2 accepts UTP and dTTP as nucleotides, as well as glucose 1-phosphate and galactose 1-phosphate as sugar phosphates. Substrate promiscuity is known for some GalUs, whereas some are very specific [39]. Indeed, it has been reported that GalUs from *S. coelicolor*, *Salmonella enterica*, *Sphingomonas elodea,* and *E. coli* are able to accept both UTP and dTTP as substrates [10,40,68,69], whereas for example the GalU from *Helicobacter pylori* is specific only for UDP [17]. It has been shown for *H. pylori* that configuration of the active site prevents a thymine from binding due to steric clashes with a methionine residue (M105) [17]. Superposition of a *Ro*GalU2 homology model, produced using SWISS-MODEL [70,71], with the crystal structure from *H. pylori* in complex with UDP-glucose (pdbID 3JUK, [17]) shows that *Ro*GalU2 does not have a methionine but a proline residue in the designated position. This observation is similar to what has been reported for *S. elodea* and *E. coli* GalUs having a proline and an alanine, respectively, at that position and also being active towards dTTP [39]. Furthermore, *Ro*GalU2 is also able to take galactose 1-phosphate as a substrate, but the enzyme is less active (only 2% residual activity), as was observed for *S. elodea* and *E. coli* GalUs [30]. It has been suggested that this less favorable binding of galactose 1-phosphate is due to the loss of the H-bond formed between the glucose 1-phosphate and the main-chain nitrogen atom of a glycine residue (Gly179 *E. coli* and Gly180 in *Ro*GalU2 homology model) [39].

**Figure 11:**
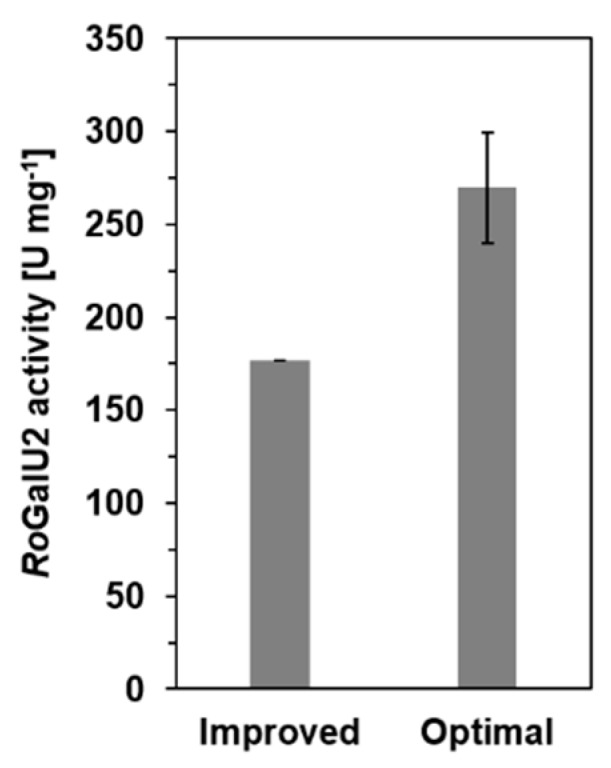
*Ro*GalU2 activity of the improved activity assay compared to an assay with optimal reaction conditions. Reaction solution for improved test contained 1.5 mM UTP, 250 mM glucose 1-phosphate, 1 mM MgCl_2_, 50 mM Hepes, pH 7.4, 1.7 µg *Ro*GalU2. Reaction was carried out at 42 °C in 1 mL scale. Reaction solution for optimal test contained 1.5 mM UTP, 250 mM glucose 1-P, 1 mM MgSO_4_, 50 mM Imidazol, pH 7.4, 1.7 µg *Ro*GalU2. Reaction was carried out 43 °C in 1 mL scale. Means with standard deviations are shown (triplicates).

Having all this in mind and the optimal conditions for *Ro*GalU2 experimentally verified, we could use this knowledge to obtain a maximum observed activity value for this UDP-glucose pyrophosphorylases in the direction of UDP-glucose formation (Figure 11). Under optimal conditions the activity of *Ro*GalU2 was 270 U mg^−1^, which is an increase in activity of about 37%. Thus this enzyme is among the most active GalUs of bacteria and might be interesting to be studied for various biotechnological applications described recently [72].

## 4. Materials and Methods 

### 4.1. Bacterial Strains, Plasmids, and Gene Synthesis

Protein sequences of the UDP-glucose pyrophosphorylases *Ro*GalU1 and *Ro*GalU2 of *Rhodococcus opacus* 1CP were taken from the NCBI accessions ANS26426 (*Ro*GalU1) and ANS26629 (*Ro*GalU2), respectively. The corresponding genes *RogalU1* (914 bp) and *RogalU2* (932 bp) used in this study were codon usage optimized to increase the expression level in *E. coli* and synthesized by Eurofins Genomics (Ebersberg, Germany) with flanking restriction sites of *Nde*I and *Not*I (GenBank accession numbers of the codon usage optimized nucleotide sequences of *RogalU1*: MN617759 and *RogalU2*: MN617760). Both genes were delivered in separate vectors (pEX-A2). They were cloned into the expression vector pET16bP (5740 bp) carrying a resistance against ampicillin, a DNA sequence that allowed the production of the GalU proteins with an *N*-terminal Histidine_10_-tag and an additional DNA sequence for the gene expression induction with isopropyl β-D-1-thiogalactopyranoside (IPTG) (see Table 3).

### 4.2. Protein Production

Transformation of *E. coli* BL21(DE3) pLysS was carried out as recommended by New England Biolabs Inc. (Ipswich, Massachusetts, USA). 

Protein production was realized in a 1 L scale in a Fernbach flask in standard LB medium (lysogenic broth: 10 g L^−1^ trypton, 5 g L^−1^ yeast extract, 10 g L^−1^ sodium chloride) with 100 mg L^−1^ ampicillin as well as 50 mg L^−1^ chloramphenicol. Expression cultures were inoculated 1:50 with an overnight pre-culture at 37 °C of *E. coli* BL21(DE3) pLysS-pET16bP-*RogalU1* or *E. coli* BL21(DE3) pLysS-pET16bP-*RogalU2* in the same medium used for the expression culture. The main culture was incubated for about 2 h at 37 °C until an OD_600_ of 0.2–0.3 could be observed. After cooling down to 20 °C, the gene expression and thus protein production started after induction with 0.5 mM isopropyl β-D-1-thiogalactopyranoside (IPTG) at an OD_600_ of 0.3–0.4. 

The large-scale protein production was performed in an analog manner in a 10 L Eppendorf bioreactor in TB autoinduction medium [74] with the same concentrations of antibiotics and inoculation culture as described above. 

After 22 h the protein production was stopped in both cases by harvesting the cells at 4 °C and 5000× *g* for 30 min. After washing with 25 mM sodium phosphate buffer, pH 7.1, pelleted cells were frozen and stored at −80 °C in portions of about 25 g of wet biomass.

### 4.3. Purification of Recombinant Proteins

For purification 25 g of pelleted cells were thawed as fast as possible in a warm water bath and mixed gently with 25 mL of a buffer containing the following compounds: 25 mM sodium phosphate buffer, pH 7.1, 100 mM sodium chloride, 1 mM magnesium chloride, 240 U of DNase I and 20 mg of lysozyme. After incubation at 30 °C for 45 to 60 min the cells were sonicated 10 times for 30 s with an intensity of 70% (Bandelin Sonoplus HD 2070, MS 72) and centrifuged at 12,000× *g* at 4 °C for 20 min. Another two centrifugation steps with the supernatant followed at 4 °C, 50,000× *g* for 30 min, each. The clear supernatant was filtered through 0.45 µm and 0.2 µm filter, respectively, before purification with an Äkta Prime Plus FPLC system with 5 mL HisTrap HP nickel column (GE Healthcare) with a flow of 5 mL min^−1^. For equilibration of the affinity chromatography column a buffer containing 25 mM sodium phosphate buffer, pH 7.1, 300 mM sodium chloride and 25 mM imidazole was used. Protein loading was performed with equilibration buffer containing 25 mM imidazole. Unspecific proteins were washed off the column with equilibration buffer containing 40 mM imidazole. Protein purification was then performed by applying a linear gradient from 40–500 mM imidazole in this buffer in a course of total 10–20 mL elution, depending on the injection volume. The protein of interest was eluted at 500 mM imidazole.

Fractions that showed UDP-glucose pyrophosphorylase activity were pooled and precipitated with 80% saturated ammonium sulfate solution. The precipitated protein was dissolved and stored in a buffer of 50 mM Hepes, pH 7.0, 100 mM sodium chloride and 1 mM magnesium chloride. Protein aliquots were stored at –80 °C for long term storage or at 4 °C for short term storage and had concentrations of about 2–8 mg mL^−1^.

Here, it needs to be mentioned that *Ro*GalU2 has a very low extinction coefficient due to the low amount of aromatic amino acids in the polypeptide chain and thus purifying by following the UV/VIS trace was somewhat difficult.

### 4.4. Renaturation of RoGalU1

Isolation of inclusion bodies and renaturation of the wrongly folded *Ro*GalU1 was realized with the Thermo Scientific Pierce Protein Refolding Kit according to the manufacturer’s instruction, but without any EDTA in the buffers or solutions. The *Ro*GalU1 concentrations for the renaturation were 1 mg mL^−1^ and 10 mg mL^−1^, respectively. 

### 4.5. Protein Determination

Determination of the protein concentration was done as described before by Bradford [75] with a bovine serum albumin (BSA) standard. Identification of the proteins was performed by determination of the molecular weight with sodium dodecyl sulfate polyacrylamide gel electrophoresis (SDS-PAGE) and coomassie staining [76]. Furthermore, the oligomeric state of the proteins was determined by size exclusion chromatography using a 24 mL Superdex 200 10/300 GL column (GE Healthcare), the manufacturers protocol and a calibration standard mix containing Ferritin, Conalbumin, Carbonic Anhydrase, Ribonuclease, and Aprotinin. Dextran Blue was used to determine the void volume of the size exclusion column. The buffer that was used for this method contained 50 mM Hepes, pH 7.0 and 1 mM magnesium chloride and was the same as used for the most characterization experiments.

### 4.6. Enzyme Activity Assay

The specific enzyme activity of *Ro*GalU1 and *Ro*GalU2 was measured only in the direction of UDP-glucose formation in a reaction volume of 1 mL. The following assay composition was used in this work mostly and thus designated as standard test. The reaction solution for initial activity measurements contained 2 mM UTP, 2 mM glucose 1-phosphate, 4 mM magnesium chloride, and 50 mM Hepes, pH 7.0. Pre-incubation of the reaction samples was done for 15 min at 30 °C and the reaction was started by addition of 30 µg of enzyme, unless otherwise indicated. Samples of 100 µL were taken after defined time points to determine the initial reaction rates. Therefore, the reaction was stopped by adding 100 µL acetonitrile and vortexing in order to denature proteins. After centrifugation for 2 min at 20,000× *g* 100 µL of the clear supernatant were used for HPLC analysis. 

In order to characterize GalU enzymes the above described standard assay was altered with respect to buffer, pH, temperature and various other additives during experimentation and to find optimal reaction conditions.

The enzyme activity is expressed as U mg^−1^. Respectively, 1 U corresponds to the formation of 1 µmol UDP-glucose per minute. This value is then referred to the amount of enzyme in the assay. 

### 4.7. Product Determination by HPLC for Specific Enzyme Activity Evaluation

HPLC measurement was performed with a Thermo Scientific Dionex Ultimate 3000 with UV/VIS detector and Macherey-Nagel EC 150/4.6 Nucleoshell HILIC column with a particle size of 2.7 µm. For determination of UDP-glucose formation an isocratic chromatography program was used with 70% acetonitrile and 30% 134 mM ammonium acetate, pH 5.35 at a flow of 1.3 mL min^−1^ and an oven temperature of 30 °C. Detection of UDP-glucose was done at a wave length of 260 nm with an injection volume of 5 µL. Calibration was carried out with appropriate concentrations of a UDP-glucose standard under the same conditions. 

For data evaluation and calculation of product formation the area of the UDP-glucose peak was used and referred to the time point of the enzyme assay to obtain an initial rate. Those rates were plotted according to the enzyme kinetic models for analysis.

The following two equations according to Michaelis–Menten (1) and Yano und Koga [50] (2);

(1) $$V\;=\;\frac{V_{\max}\;\cdot {\;c}_{S}}{K_{m}\;+{\;c}_{S}}$$

(2) $$V\;=\;\frac{V_{\max}\;\cdot {\;c}_{S}}{K_{m}\;+{\;c}_{S}\;+\;\frac{c_{S}^{3}}{K_{i}^{2}}}$$

have been used. Equation (1) was used for non-limiting conditions and 2 for the cases of substrate inhibition. 

## 5. Conclusions

The biochemical characterization of UDP-glucose pyrophosphorylases has been reported for several organisms. Here, we described the first characterization of a UDP-glucose pyrophosphorylase from a *Rhodococcus* strain that contained two isogenes coding for GalUs. Based on phylogenetic analyses and activity data obtained it seems obvious that *Ro*GalU2 represents the metabolically active enzyme, whereas the role of *Ro*GalU1 remains enigmatic and needs to be investigated. Furthermore, the activity of *Ro*GalU2 is higher than that of some other reported GalUs, but the use of conventional kinetic models is limited. Therefore, further biochemical investigation will be necessary. In addition, this is the first report of the use of magnesium sulfate as metal cofactor. The sulfate salt of magnesium was able to double the activity of *Ro*GalU2. A maximum activity of *Ro*GalU2 of about 270 U mg^−1^ was determined which renders it a candidate for further biocatalytic investigations.

## Figures and Tables

**Table 1:** Kinetic properties of *Ro*GalU2.

Varied Substrate	UTP ^1^	Glucose 1-Phosphate ^1^	
conc. [mM]	0.01–5	0.05–10	0.05–500
apparent *K*_m_ [mM]	0.51	0.9	150
*V*_max_ [U mg^−1^] ^2^	177	6.75	-
*V*_max,ob_ [U mg^−1^] ^3^	122	6.68	119
apparent *k*_cat_ [s^−1^]	96.2	3.7	65
*K*_I_ [mM]	2.61	-	225
*k*_cat_/*K*_m_ [µM^−1^ s^−1^]	0.19	0.004	0.0004

^1^ Assay mixture contained substrates at varied concentrations given whereas the other substrate was set constant at 250 mM glucose 1-phosphate or 1.5 mM UTP, respectively, 1 mM MgCl_2_, 50 mM Hepes buffer, pH 7.4, 1.7 µg *Ro*GalU2. Reaction was carried out at 42 °C in 1 mL volume. All experiments were performed at least in triplicates. Fits were according Michaelis–Menten or Yano and Koga [50]. ^2^ The specific activity was obtained from fitting the data. ^3^ The maximal observed activity was taken as a read out from the plot.

**Table 2:** Kinetic properties of UDP-glucose pyrophosphorylases used for the phylogenetic analysis. Units are given in brackets in the table head, unless otherwise indicated.

Organism and Protein	*V*_max_ [U mg^−1^]		*K*_m_ [mM]		*k*_cat_ [s^−1^]		c [mM]		Ref.
	UTP	G1P	UTP	G1P	UTP	G1P	UTP	G1P	
*R. opacus* 1CP GalU2	177	6.75^1^ 119^2^	0.51	0.9^1^ 150^2^	96.2	3.7^1^ 65^2^	0.01–5	0.05–500	This study
*E. coli* K-12 GalU	340		0.17	0.035	187		2	2	[19]
*E. coli* K-12 GalF	0.015		0.36	0.52	0.008		2	2	[19]
*M. tuberculosis* H37Rv GalU	2.5–2.7^3^ 9.8 U^4^	5.8 U^4^	0.1^3^ 0.012^4^	0.13^3^ 0.045^4^	93.44^4^	55.03^4^	0.001–0.01^4^	0.008–0.05^4^	[51,13]
*E. amylovora* CFBP 1430 GalU	14.4	32.5	0.027	0.007	7.9	17.2	0.006–0.1	0.003–0.1	[30]
*S. coelicolor* A3 (2) GalU	270		>10	0.06	149		≤20	n.s.	[10]
*B. bifidum* PRL2010 GalU	13		0.042	0.098	12		0–2	0–2	[59]
*X. capestris* pv. *campestris* str. 8004 GalU	60–90		0.21	0.06	40		n.s.	0-8	[31]
*X. axonopodis* pv. *citris* str. 306 GalU	60–90		0.11	n.s.	29		n.s.	n.s.	[31]
*S.thermophilus* LY03 GalU	0.2		n.s.	n.s.	0.11		1.25	1	[32]

^1^ for glucose-1-phosphate concentrations up to 10 mM; ^2^ for glucose-1-phosphate concentrations between 10–500 mM; ^3^ [51]; ^4^ [13]; n.s.: not specified; The *K*_m_ and *k*_cat_ values in case of *Ro*GalU2 are apparent values, see Table 1.

**Table 3:** Strains, plasmids, and primers used in this study.

Sample	Relevant Characteristics	Source, Reference
**Strains**		
*R. opacus* 1 CP	Benzoate^+^, 4-hydroxybenzoate^+^, 3-chlorobenzoate^+^, phenol^+^, 4-chlorophenol^+^, 2,4-dichlorophenol^+^, 2-chlorophenol^+^, 3-methylphenol^+^, 4-methylphenol^+^, phthalate^+^, isophthalate^+^, *n*-alkanes^+^ (C10-C16), styrene^+^	[73,64]
*E. coli* DH5α	fhuA2 Δ(argF-lacZ) U169 phoA glnV44 Φ80 Δ(lacZ)M15 gyrA96 recA1 relA1 endA1 thi-1 hsdR17	NEB ^1^
*E. coli* BL21(DE3) pLysS	fhuA2 [lon] ompT gal (λ DE3) [dcm] ∆hsdS λ DE3 = λ sBamHIo ∆EcoRI-B int::(lacI::PlacUV5::T7 gene1) i21 ∆nin5	NEB ^1^
**Plasmids**		
pEX-A2	multiple cloning site, Lac-Promoter, pUC origin, Amp^r^	Eurofins ^2^
pET16bP	pET16b with additional multiple cloning site; allows production of recombinant proteins with N-terminal Histidine_10_-Tag and gene expression induction with IPTG	Wehmeyer ^3^
pEX-A2-*RogalU1*	pEX-A2 vector with recombinant UDP-glucose-pyrophosphorylase gene 1 of *R. opacus* 1 CP (*RogalU1*) 914 bp, NCBI protein accession: ANS26426	This study
pEX-A2-*RogalU2*	pEX-A2 vector with recombinant UDP-glucose-pyrophosphorylase gene 2 of *R. opacus* 1 CP (*RogalU2*) 932 bp, NCBI protein accession: ANS26629	This study
pET16bP-*RogalU1*	pET16bP vector with recombinant UDP-glucose-pyrophosphorylase gene 1 of *R. opacus* 1 CP (*RogalU1*)	This study
pET16bP-*RogalU2*	pET16bP vector with recombinant UDP-glucose-pyrophosphorylase gene 2 of *R. opacus* 1 CP (*RogalU2*)	This study
**Primer**		
pET16bP-fw	5’ - CATCACAGCAGCGGCCATATCGAAG - 3’	This study
pET16bP-rev	5’ - CAGCTTCTTTTCGGGCTTTGTTAG - 3’	This study

^1^ New England Biolabs Inc. or ^2^ Eurofins Genomics as a commercial source. ^3^ personal communication by U. Wehmeyer.
